# Combining Non Selective Gas Sensors on a Mobile Robot for Identification and Mapping of Multiple Chemical Compounds

**DOI:** 10.3390/s140917331

**Published:** 2014-09-17

**Authors:** Bennetts Victor Hernandez, Erik Schaffernicht, Victor Pomareda, Achim J. Lilienthal, Santiago Marco, Marco Trincavelli

**Affiliations:** 1 Applied Autonomous Sensor Systems, Örebro University, Fakultetsgatan 1, 70182 Örebro, Sweden; E-Mails: erik.schaffernicht@oru.se (E.S.); achim.lilienthal@oru.se (A.J.L.); marco.trincavelli@oru.se (M.T.); 2 Signal and Information Processing for Sensing Systema, Institute for Bioengineering of Catalonia, Baldiri Reixac 4-8, 08028-Barcelona, Spain; E-Mails: vpomareda@ibecbarcelona.eu (V.P.); smarco@ibecbarcelona.eu (S.M.); 3 Departament d'Electròica, Universitat de Barcelona, Martí i Franqués 1, 08028-Barcelona, Spain

**Keywords:** environmental monitoring, gas discrimination, gas distribution mapping, service robots, open sampling systems, PID, metal oxide sensors

## Abstract

In this paper, we address the task of gas distribution modeling in scenarios where multiple heterogeneous compounds are present. Gas distribution modeling is particularly useful in emission monitoring applications where spatial representations of the gaseous patches can be used to identify emission hot spots. In realistic environments, the presence of multiple chemicals is expected and therefore, gas discrimination has to be incorporated in the modeling process. The approach presented in this work addresses the task of gas distribution modeling by combining different non selective gas sensors. Gas discrimination is addressed with an open sampling system, composed by an array of metal oxide sensors and a probabilistic algorithm tailored to uncontrolled environments. For each of the identified compounds, the mapping algorithm generates a calibrated gas distribution model using the classification uncertainty and the concentration readings acquired with a photo ionization detector. The meta parameters of the proposed modeling algorithm are automatically learned from the data. The approach was validated with a gas sensitive robot patrolling outdoor and indoor scenarios, where two different chemicals were released simultaneously. The experimental results show that the generated multi compound maps can be used to accurately predict the location of emitting gas sources.

## 1. Introduction

Emission monitoring of gaseous substances in industrial processes is of significant importance for governmental agencies due to its economical and environmental implications [1]. Due to their versatility and adaptability, gas sensitive mobile robots can make important contributions to this task. For example, mobile robots can be used as autonomous systems that can adaptively collect measurements at a denser spatial and temporal granularity, compared to stationary sensor networks. Contrary to human operators, robots can be placed in hazardous environments and they can perform repetitive measurement processes for prolonged periods of time.

The measurements collected with a mobile platform can be presented conveniently to human operators through a set of gas distribution maps. Gas Distribution Modeling (GDM) is the process of learning a truthful representation of the observed gas distribution from a set of spatially and temporally distributed measurements of relevant variables, foremost gas concentration but also wind, pressure and temperature [2].

GDM is a challenging task due to both the complex mechanisms that govern gas dispersion and the limitations of chemical sensing. Gas dispersion is governed by three physical phenomena, namely turbulence, advection and diffusion. In environments where the flow regime is characterized by a high Reynolds number [3], the airflow is turbulent and therefore characterized by chaotic eddies and vortices at different scales. Due to the slow diffusion process, gas dispersion is mostly governed by turbulence and advection. Therefore, the structure of the gas distribution on a short timescale is irregular and highly unpredictable and, under general conditions, no functional shape of the gas distribution can be assumed a priori.

Additional challenges are introduced by the limitations of currently available chemical sensors. First of all, conventional chemical sensing technologies are *in situ*. This means that a direct interaction between the sensor surface and the compound molecules is required and each measurement provides information about a small spatial region of a few centimeters around the sensor. In comparison, *remote* gas sensing technologies, such as Tunable Diode Laser Absorption Spectroscopy (TDLAS) sensors [4], can provide information about the space several meters away from the position of the sensor itself. However, remote sensing devices are considerably more expensive than *in situ* sensors and furthermore, they report spatially unresolved concentration measurements. This means that measurements are reported as integral values, with no information regarding the spatial distribution of the sensed concentrations [5].

A second limitation of some *in situ* sensing technologies, for example metal oxide sensors, is their partial selectivity. This means that a sensor can respond to a variety of compounds and not only to a single target compound as it is often claimed by the manufacturer. In order to mitigate this problem, current GDM algorithms rely on the assumption that only a single chemical compound is released at a given time.

In this work, we present a set of algorithms to produce gas distribution models of multiple heterogeneous chemical compounds. The identity of the measurements is determined by using an open sampling system, composed of an array of metal oxide sensors, and a probabilistic gas discrimination algorithm. The distinctive characteristic of this algorithm is that the class posteriors are determined according to the particular selectivity strengths of the sensor array [6]. To generate calibrated gas distribution models of multiple compounds, we use an improved version of the Multi Compound (MC) Kernel DM+V algorithm, originally presented in [7]. MC Kernel DM+V uses the class posterior probabilities, computed by a given classifier, and concentration readings, from a Photo Ionization Detector (PID), to create independent gas distribution maps for each target compound.

In the improved MC Kernel DM+V algorithm presented in this work, the gas distribution models are generated by considering the spatial extrapolation of the class posteriors in order to filter out incorrect predictions from the gas discrimination algorithm. In addition, the correction factors of the PID which are provided by the manufacturer, are incorporated in the computation of the distribution maps. In this way, the predictions made by the gas distribution maps can be considered as calibrated concentration values. The problem of parameter selection for multi compound gas distribution mapping is addressed in this paper with an approach that allows to automatically select the parameters of the algorithm from the collected data. The algorithm was validated not only in an indoor environment, but also in an outdoor location, where two different chemical compounds were released at the same time. The results suggest that the models predicted by MC Kernel DM+V can be used to localize emitting gas sources, which is a crucial feature for emission monitoring applications.

This paper is structured as follows: related work is discussed in Section 2 while the MC Kernel DM+V algorithm is presented in Section 3. In Section 4, the problem of gas discrimination in open environments is discussed. The experimental set-up is described in Section 5 and the results are presented in Section 6. We conclude this paper in Section 7, where we discuss the results and make suggestions for future work.

## 2. Related Work

The existing algorithms to derive gas distribution models can be divided in *model based* and *model free* approaches. *Model based* algorithms assume that the spatial distribution of gas concentrations can be explained by an underlying mathematical model, which is regulated by a set of functional parameters. This family of algorithms are also often used to simulate gas dispersion in large scale areas, up to hundreds of kilometers [8].

*Model based* algorithms, such as Gaussian shaped plumes [9], can be used for gas source localization [10] and large scale release rate prediction of airborne chemicals [11]. However, these approaches generate overly simplistic models since they assume uniform and constant laminar windflow patterns.

More complex algorithms can create sophisticated gas distribution maps by using puff models [12], particle models [13] or by incorporating Computational Fluid Dynamics (CFD) [14]. However, tractability becomes an issue, since precise knowledge is required about boundary conditions, which is rarely available. Simplifying assumptions can be made (e.g., unidirectional wind fields, flat terrains), but they lead to unrealistic models and thus, inaccurate predictions.

Alternatively, GDM can be addressed by using *model free* approaches, as the algorithm proposed in this work. These algorithms do not make strong assumptions about the underlying functional form of the gas distribution but rather treat sensor measurements as random variables and derive statistical representations of the observed gas dispersion. However, a common assumption made by these algorithms is that only one chemical compound is present in the environment and thus, there is no information regarding the identity of the gases in the distribution maps. *Model free* algorithms have been mostly used in small scale applications, for example, to generate distribution maps in indoor locations and outdoor courtyards of a few square meters [15–17].

The work of Ishida [18] includes probably the first approach to create *model free* gas distribution maps. The authors generated a discrete gas distribution representation where the concentration at each measurement point was modeled by the average of the sensor response measured during five minutes. Hayes and co-workers proposed in [19] an algorithm in which two dimensional histograms were used to represent the spatial distribution of water vapour. The bins in the histograms count the number of *odor hits* registered at a given location in the exploration path. An odor hit is counted if the sensor response exceeded a threshold value.

Lilienthal and Duckett [20] introduced the Kernel DM gas distribution modeling algorithm, which performs spatial integration of the sensor measurements with a radially symmetric 2-D Gaussian function. The gas distribution model generated by this algorithm is represented in the form of a grid map, in which a mean concentration value is estimated for each cell. More recently, Lilienthal and co-authors presented in [21] a novel GDM algorithm that rather than modeling the spatial distribution of average gas concentrations, it models the spatial distribution of *detection* events of a given target chemical compound. The algorithm is based on the Bayesian Inference framework and models the likelihood of *detection* events at a given query location. The advantage of this method is that readings from sensors with different sensing principles can be integrated in the computation of the distribution maps.

A shortcoming of the methods discussed above is that no estimation is given about the fluctuations of the gas concentration. The spatial distribution of the gas fluctuations can convey useful information for, e.g., environmental monitoring. For example, areas of high concentration fluctuations can be correlated to the location of an emitting gas source [22]. Furthermore, the estimation of the predictive variance provides several advantages for gas distribution modeling for example, it allows to evaluate the model quality in terms of the data likelihood [23]. In addition, the predictive variance can be used in sensor planning algorithms to suggest new measurement locations [24].

There are several examples of algorithms that provide an estimation of the gas fluctuations. In [2], the authors proposed the Kernel DM+V algorithm, which estimates the spatial distribution of the mean concentration and the predictive variance (*i.e.*, gas fluctuations) in the form of discretized grid maps. At the core of the algorithm is the Nadaraya-Watson estimator with a Radial Basis Function (RBF) kernel [25], which is applied twice in an intertwined manner, once for the estimation of the predictive mean and once for the estimation of the predictive variance.

Stachniss and co-authors presented an approach based on Gaussian Process Mixture (GPM) models [15]. The proposed method allows to represent the rather smooth “background signal” and the areas of high concentration by using different components of the GPM. The components of the mixture model and a gating function, that decides to which component a data point belongs, are learned using Expectation Maximization (EM). Blanco and co-authors presented in [26] a Bayesian approach to generate mean and variance gas distribution models in 2D environments. The authors used a sparse implementation of a Kalman filter that allowed to update the models and make predictions on-line. This algorithm was validated with a dataset collected with a gas sensitive mobile robot inside a sealed room.

An implicit assumption made by the above mentioned algorithms is that only one chemical compound is present in the environment. However, in practical applications this assumption rarely holds. In order to solve a multi compound GDM problem, it is necessary to incorporate a stage of gas discrimination, where the identity of the substances is estimated and used in the subsequent distribution mapping task.

Gas discrimination can be carried out with devices that combine arrays of partially selective gas sensors and pattern recognition algorithms. These devices are commonly called electronic noses (e-noses) [27]. Sensor arrays can be constructed with sensors based on different technologies such as carbon nanotubes, electrochemical cells, polymers and metal oxide semiconductors [28]. While the selection of the sensors is application specific, metal oxide (MOX) sensors are the most widely used sensors in e-nose applications due to their wide commercial availability, fast response times and high sensitivity. MOX sensors are conductometric, which means that the interaction of a target analyte with the sensing surface causes a change in the sensor's resistance.

In outdoor environments, gas discrimination poses a challenging task since the environmental variables that affect the gas dispersion mechanisms cannot be controlled. In these scenarios, the sensing devices are directly exposed to environmental conditions and therefore, the gas concentration measurements are constantly fluctuating. Sensing devices directly exposed to the surrounding environment are typically referred to as Open Sampling Systems (OSS). Gas discrimination has mainly been studied under laboratory conditions, where factors such as temperature and humidity are tightly controlled and the sensors are exposed to constant concentrations, allowing them to reach steady response levels. Under these conditions, applications such as food spoilage [29], wine classification [30] and bacteria detection [31] have been successfully performed.

A thorough investigation of the challenge of gas discrimination with OSS was presented by Vergara *et al.* [32,33]. The authors constructed a test-bed that consisted of a 2.5 *m* × 1.2 *m* × 0.4 *m* wind tunnel and a set of 9 OSS nodes that can be placed at different distances. Gas discrimination was performed for up to 10 different chemical compounds under a variety of wind flow regimes. The authors concluded that the performance of the system is heavily influenced by the wind conditions. In order to have a robust performance, the system should be trained under all the possible environmental conditions, which due to practical reasons, is not feasible. Thus, the training of the classifier has to be performed under a subset of these conditions.

Trincavelli and co-authors investigated the use of mobile robots for gas discrimination [34–36]. Among several contributions, the authors evaluated the possibility of performing gas discrimination using different classification algorithms and feature extraction methods, using the transient edges of the sensor response. In their work, different arrays of MOX sensors were mounted on robotic platforms and the corresponding evaluation experiments were performed in a wide range of experimental scenarios such as an unventilated room, different indoor corridors and an outdoor courtyard. In the test scenarios, two different analytes were released from mock up emitting sources.

With the exception of [7], which is a predecessor of this paper, to the authors best knowledge the only work that addresses GDM of multiple chemical compounds was presented in [16]. The authors used a mobile robot equipped with an e-nose to collect data indoors and outdoors where two different chemical substances where placed, either separated by a physical barrier or separately in independent experiments. The authors successfully generated mean distribution maps for each of the substances using the algorithm proposed in [20] and a classifier to decide to which map the measurement exclusively contributes. However, gas fluctuations were not modeled. Moreover, in [16], a significant amount of measurements, and thus information, was discarded using a threshold, which rejected low concentration measurements. Low concentration measurements can convey useful information for GDM since they can be used to model the absence of gas in the environment.

## 3. The MC Kernel DM+V Algorithm

**Figure 1.**
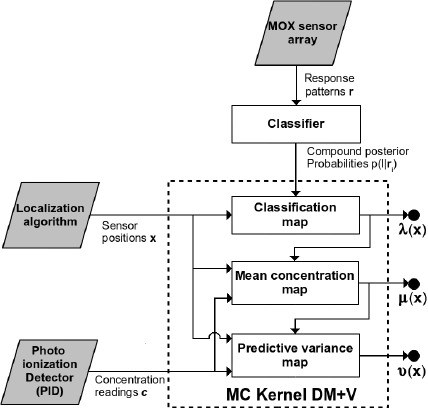
MC Kernel DM+V block diagram.

The Multi Compound (MC) Kernel DM+V is a *model free* GDM algorithm that generates a statistical representation of the spatial distribution of multiple chemical compounds. The only assumptions made are that localized gas concentration measurements are acquired with a non selective sensor and that the identity of the measurements is given as a set of posterior probabilities, computed by an external gas discrimination module. A block diagram of the algorithm is shown in Figure 1. The gas concentration measurements *c* are acquired with a PID, and the information regarding the gas identity is provided in the form of compound posterior probabilities *p*(*l*|**r**), computed using the response pattern **r**, acquired with an array of partially selective sensors. Both *c* and *p*(*l*|**r**) are associated to a measurement location x. The presented MC Kernel DM+V algorithm does not consider the uncertainty in the computation of the measurement locations x.

For each identified compound *l* (*l* ∈ *L*), MC DM+V computes three maps of the spatial distribution and concentration fluctuation of gaseous patches at a given exploration area. The mean and variance maps (*μ_l_*(**x**) and *ν_l_*(**x**) respectively) can be seen as a snapshot of the gas distribution from which at each query location x, predictions of the concentration level and its fluctuations can be estimated. The classification maps (*λ_l_*(x)) model the likelihood of detecting compound *l* at a location x.

The computation of the maps is carried out in a sequential way from a set of *n* measurements. The exploration area is discretized in a grid of cells and the classification maps *λ_l_*(x) are computed first, followed by the predictive mean maps *μ_l_*(x) and predictive variance maps *ν_l_*(x). The classification maps *λ_l_*(x) are computed by spatially extrapolating the localized posteriors *p*(*l*|**r***_i_*) using the following equations:

(1a) $$\Omega^{\left(k\right)}=\sum_{i=1}^{n}N(|{\text{x}}_{i}-{\text{x}}^{\left(k\right)}|,\sigma )$$

(1b) $$P_{l}^{\left(k\right)}=\sum_{i=1}^{n}N(|{\text{x}}_{i}-{\text{x}}^{\left(k\right)}|,\sigma )\cdot p(l|{\text{r}}_{\text{i}})$$

(1c) $$\alpha^{\left(k\right)}=1-e^{\frac{-{\left(\Omega^{\left(k\right)}\right)}^{2}}{{\left(\sigma \cdot \sqrt{2\pi }\right)}^{-2}}}$$

(1d) $$\lambda_{l}^{\left(k\right)}=\alpha^{\left(k\right)}\cdot \frac{P_{l}^{\left(k\right)}}{\Omega^{\left(k\right)}}+\{1-\alpha^{\left(k\right)}\}\cdot p_{l0}$$

Equations (1a) and (1b) are intermediate computations and correspond to a weight map and the weighted compound posteriors respectively. 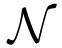 is a uni-variate Gaussian weighting function that models the importance of the posteriors *p*(*l*|**r_i_**) according to the distance to the cell centers x^(^*^k^*^)^. The parameter *σ* (*i.e.*, the Kernel Bandwidth) controls the smoothing level of 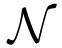 and thus, a proper selection of *σ* determines the predictive capabilities of the model.

The confidence map *α*^(^*^k^*^)^ provides an estimate of the confidence in the predictions at a given cell *k*. When *α*^(^*^k^*^)^ is close to 1, the estimations were computed using a large number of measurements recorded close to the center of cell *k*, while a value close to 0 means that only a very few or no measurements were available to compute the posterior estimation at cell *k*.

The final computation of $\lambda_{l}^{\left(k\right)}$ is given by Equation (1d), where *p_l_*_0_ is a prior assumption on the gas identity. When no other information is available, *p_l_*_0_ can be set to $\frac{1}{\text{L}}$. It can be noticed that, when there is a high number of measurements near a cell (*k*) (*i.e.*, *α*^(^*^k^*^)^ ≃ 1), the predictions will be largely determined by the weighed posteriors in Equation (1b) while, when few measurements area available close to *k*, a value close to the prior *p_l_*_0_ will be predicted by the classification map $\lambda_{l}^{\left(k\right)}$.

The predictions of the classification maps are subsequently integrated in the computation of the mean and variance maps by using the following *maximum a posteriori* function:

(2) ψ(xi)={1λl(k)(xi)>λj(k)(xi)|l,j∈L0otherwise

*ψ*(**x***_i_*) evaluates the predictions made by the maps *λ_l_* at each measurement location x*_i_*. This implies that, instead of considering individual instantaneous compound posteriors, the identity of neighboring measurements are as well considered in *λ_l_*. In this way, erroneous predictions in the gas identification are filtered out and they do not contribute to the computation of *μ_l_* and *ν_l_*. Equation (2) implicitly assumes that *λ_l_* is sufficiently stable over time. The function *ψ_li_* returns an *L* × 1 vector in which 1 is assigned to the compound predicted with the highest prior, and zero is assigned otherwise. Thus, the mean concentration maps (*μ_l_*) are computed as follows:

(3a) $$C_{l}^{\left(k\right)}=\sum_{i=1}^{n}N(|{\text{x}}_{i}-{\text{x}}^{\left(k\right)}|),\sigma )\;\cdot \;c_{i}\;\cdot \;\xi_{l}\;\cdot \;\psi_{l}({\text{x}}_{i})$$

(3b) $$\mu_{l}^{\left(k\right)}=\alpha^{\left(k\right)}\;\cdot \;\frac{C_{l}^{\left(k\right)}}{\Omega^{\left(k\right)}}+\{1-\alpha^{\left(k\right)}\}\;\cdot \;c_{l0}$$

where the parameter *ξ_l_* corresponds to the *correction factor* for compound *l*. This parameter is related to one of the assumptions made by the algorithm, which is that the concentration measurements are given by a non specific gas sensor. In the case of a PID, the device is calibrated with a reference gas (e.g., isobutylene) and the manufacturer provides a table with *correction factors* for different compounds. Thus, once the chemical has been identified, the measurement reported by the device has to be multiplied by the corresponding *correction factor* to obtain calibrated concentration measurements. Similarly to the classification maps, *α*^(^*^k^*^)^ in Equation (3b) balances between the weighted concentration values in Equation (3a) and a prior assumption on the gas concentration *c_l_*_0_ for each compound *l*.

Using a prior assumption on the variance *υ_l_*_0_ for each compound, the variance maps can be computed as follows:

(4a) $$V_{l}^{\left(k\right)}=\sum_{i=1}^{n}N(|{\text{x}}_{i}-{\text{x}}^{\left(k\right)}|,\sigma )\;\cdot \;{\left(c_{i}\;\cdot \;\xi_{l}-\mu_{l}^{\left(k\right)}({\text{x}}_{i})\right)}^{2}\;\cdot \;\psi_{l}({\text{x}}_{i})$$

(4b) $$\nu_{l}^{\left(k\right)}=\alpha^{\left(k\right)}.\frac{V_{l}^{\left(k\right)}}{\Omega^{\left(k\right)}}+\{1-\alpha^{\left(k\right)}\}\cdot \upsilon_{l0}$$

where Equation (4a), computes the weighted square error between the corrected concentration measurements and the predictions made by the mean concentration maps.

## 4. Gas Discrimination with Open Sampling Systems

**Figure 2.**
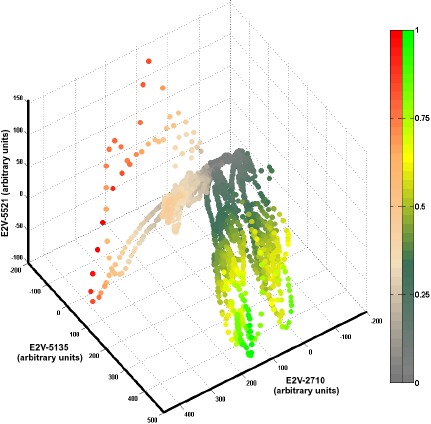
Feature space for a two class gas discrimination problem. Each data point represents the instantaneous concentration measurement acquired with a sensor array composed of 3 sensors. The color shades denote the normalized concentration level. Green shades are assigned to ethanol samples, while red shades are assigned to propanol samples. The axes units represent the baseline corrected sensor responses.

Before presenting the experimental evaluation of the MC Kernel DM+V, it is important to discuss the particular characteristics of datasets collected with OSS. Figure 2 illustrates the feature space plot of a dataset collected with a mobile robot equipped with a 3-sensor MOX array. The instantaneous responses are considered as features and the data was collected indoors where two sources of different gases (namely ethanol and propanol) were placed in six independent experiments, three for each substance. Brighter color shades are assigned to high concentration measurements, while low concentrations are plotted in gray tones.

It can be noticed in Figure 2 that the dataset is not evenly represented with respect to the different gas concentrations. High concentration measurements are sparse, while most of the data lies in the low to mid concentration regions. A density based classification algorithm tends to assign higher class posterior probabilities to measurement points that lie inside densely populated regions, and low posterior probabilities for sparsely represented concentrations. In addition, it can be seen that there is a high degree of separability at high concentration levels, while for lower concentrations, the classification problem becomes ambiguous as expected. Thus, the concentration measurements convey useful information for the gas discrimination problem.

### 4.1. Incorporating Concentration Information in the Discrimination Process

Given these observations, in [6] we proposed an approach that tends to assign higher posteriors to measurements that lie in the feature space regions of high concentration, and low posteriors to measurements which correspond to the overlapping areas in the feature space at low concentrations. In addition, low concentration measurements close to the reference response level of the sensors are assigned to a third implicit class *l_a_* that denotes the absence of chemicals (*i.e*., clean air).

The gas discrimination approach does not require calibrated concentration measurements as inputs and thus, a rough indicator of the concentration level is computed using the instantaneous measurements acquired with the OSS. The instantaneous sensor responses can be used as indicators of the concentration level since over a certain concentration range the logarithm of the change in resistance of a MOX sensor is linearly proportional to the logarithm of the gas concentration [37].

For a given array of *D* MOX sensors, the uncalibrated concentration indicator *I_c_* for a measurement response **r***_i_* can be computed as follows:

(5) $$I_{c}({\text{r}}_{i})=\underset{r_{i}^{\left(j\right)}\in \;{\text{r}}_{i}}{\max}\left(r_{i}^{\left(1\right)},r_{i}^{\left(2\right)},\ldots ,r_{i}^{\left(D\right)}\right)$$

The class posterior *p*(*l*|r) is estimated by coupling the pairwise probabilities between the target chemical compounds (*P_l_*__1_∨_*_l_*__2__ and *P_l_*__2_∨_*_l_*__1__) and the pairwise probabilities between each of the compounds and the rejection class *l_a_* (*P_l_*__1_∨_*_l_*__*a*__ and *P_l_*__2_∨_*_l_*__*a*__). There is no specific requirement regarding the classification method to compute *P_l_*__1_∨_*_l_*__2__ and *P_l_*__2_∨_*_l_*__1__ and thus, any algorithm can be chosen as long as *P_l_*__2_∨_*_l_*__1__ = 1 − *P_l_*__1_∨_*_l_*__2__. In the implementation presented in this work, we opted for a Mixture of Gaussians Classifier (MoGC). A MoGC is a mixture model [38] that computes the data densities *p*(**x**|*γ*) by a linear combination of *γ* Gaussian functions weighted by mixing coefficients.

**Figure 3.**
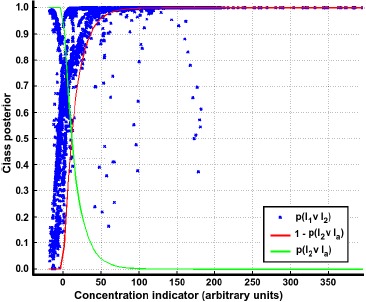
Pairwise class probability plots. The blue markers denote *p*(*l*_1_ ∨ *l*_2_) and the red and green lines are the pairwise probabilities *p*(*l*_2_ ∨ *l_a_*) and 1 − *p*(*l*_2_ ∨ *l_a_*) respectively.

To illustrate the process for determining *P_l_k__*_∨_*_l_a__*, a plot of *I_c_ vs. p*(*l*_1_ ∨ *l*_2_) is shown in Figure 3. It can be noticed that, at low concentrations, *P_l_*__1_∨_*_l_*__2__ shows strong fluctuations, which is an indication of the high uncertainty close to the sensor's reference values. Thus, a model for *P_l_x__*_∨_*_l_a__* can be learned in such a way that the confidence in the prediction smoothly increases as a function of *I_c_*.

The pairwise class probabilities *P_l_x__*_∨_*_l_a__* can be thus modelled as an exponential function with the concentration indicator *I_c_* as the input variable as follows:

(6) Plk∨la(Ic)=1−e−βkIc|k=1,2

where the functional parameters *β_k_*, determine the rate of change in the class probability predictions. The functional parameters *β_k_* can be individually learned from the data by dividing the training dataset according to their labels *l_k_* and using the pairwise probabilities between the compounds (e.g., *P_l_*__1_∨_*_l_*__2__) as target variables.

The final computation of the class posteriors *p*(*l*|**r**) is obtained by coupling the binary class probabilities. In the implementation presented in this work, we used the algorithm proposed by Hastie *et al.* in [39,40], which frames the estimation of the posterior probability as the minimization of the Kullback-Leibler (KL) distance between the pairwise estimates and the true distributions. For a two class discrimination problem, *p*(*l*|**r**) is computed as follows:

(7a) $$p(l_{1}|\text{r})=1-\frac{2\;\cdot \;P_{l_{1}\vee l_{a}}\;\cdot \;P_{l_{1}\vee l_{2}}-2\;\cdot \;P_{l_{1}\vee l_{a}}+2}{P_{l_{1}\vee l_{a}}\;\cdot \;P_{l_{1}\vee l_{2}}-P_{l_{2}\vee l_{a}}\;\cdot \;(P_{l_{1}\vee l_{a}}+P_{l_{1}\vee l_{a}}-1)+2}$$

(7b) $$p(l_{2}|\text{r})=\frac{2\;\cdot \;P_{l_{2}\vee l_{a}}\;\cdot \;P_{l_{1}\vee l_{2}}-2}{P_{l_{2}\vee l_{a}}-P_{l_{1}\vee l_{a}}\;\cdot \;(P_{l_{2}\vee l_{a}}-P_{l_{1}\vee l_{2}})-P_{l_{2}\vee l_{a}}\;\cdot \;P_{l_{1}\vee l_{2}}+2}+1$$

(7c) $$p(l_{a}|\text{r})=1-\frac{2\;\cdot \;P_{l_{1}\vee l_{a}}+2\;\cdot \;P_{l_{2}\vee l_{a}}-2\;\cdot \;P_{l_{1}\vee l_{a}}\;\cdot \;P_{l_{2}\vee l_{a}}}{P_{l_{2}\vee l_{a}}+P_{l_{1}\vee l_{2}}\;\cdot \;(P_{l_{1}\vee l_{a}}-P_{l_{2}\vee l_{a}})-P_{l_{1}\vee l_{a}}\;\cdot \;P_{l_{2}\vee l_{a}}+2}$$

## 5. Experimental Setup

**Figure 4.**
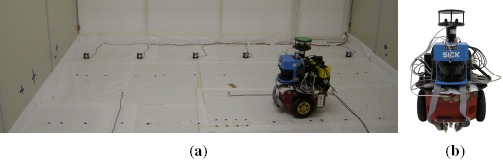
**(a)** Robot Arena. **(b)** Pioneer P3-DX platform.

**Figure 5.**
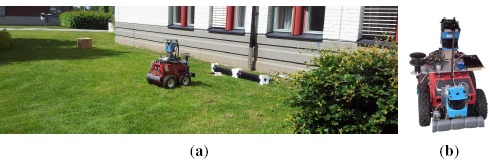
**(a)** Outdoor courtyard. **(b)** ATRV-JR platform.

The experimental configurations used to validate the proposed algorithm are listed in Table 1. Data acquisition was performed in two locations, namely a robot arena (*i.e.*, a closed room) and an outdoor courtyard (Figures 4a and 5a respectively). The experimental conditions allow to evaluate the proposed algorithm under different environmental and wind flow regimes.

Ethanol, propanol and acetone vapors were used as target chemicals. These analytes are invisible in air and in small quantities, harmless to humans. With boiling points of 78.4 °C, 82.5 °C and 56 °C respectively, they evaporate quickly at room temperature and, since they are heavier than air propagate at ground level.

The robot arena (Figure 4a) comprises an area of 5 *m* × 5 *m* × 2 *m* and while no artificial airflow was induced, a weak circulating airflow field (0.01 − 0.03 *m*/*s*) was formed in the room by natural convection. During the experimental trials, the gaseous analytes were released from two tubes at a constant 0.2 *l*/*min* flow rate.

Data was collected autonomously with a robotic platform that was programmed to follow a pre-defined trajectory and data was collected at a 4 *Hz* sampling frequency. A total of 6 experiments were conducted with a single gaseous source, 3 for ethanol and 3 for propanol. In addition, 6 dual source trials were conducted with ethanol and propanol sources, separated by different distances, as shown in Table 1. Each experiment had a duration of approx. 1800 *s* and the robot was driven at a speed of 0.04 *m/s*.

Due to size restrictions imposed by the test scenario, a compact, maneuverable robot is desired. Therefore, a Pioneer P3-DX (from MobileRobots) was used to collect data. The P3-DX (shown in Figure 4b), was equipped with an e-nose of six commercially available *MICS E2V* MOX sensors and a photo ionization (PID) gas detector (ppbRAE 3000, RAE Systems). The PID detector provides calibrated readings of the gas concentration and the responses of the e-nose's sensors enable gas detection. In addition, the robot was equipped with a frontal SICK LMS200 laser scanner for localization.

A second set of experiments was conducted in an outdoor courtyard (Figure 5a). An ATRV-JR robot (Figure 5b) was programmed to follow a random trajectory inside a 9 *m ×* 5 *m* area, where a fan was placed on the ground to generate an advective airflow and the target analytes were released from an open plastic container, using a *bubbler* to facilitate evaporation. Along the exploration trajectory, data was collected at a 4 *Hz* sampling rate. 2 trials with single emitting source, one for each analyte and a trial with two emitting sources were conducted in this scenario, as listed in Table 1. At each trial, the robot was driven at a speed of 0.12 *m/s* and each experiment had a duration of approx. 5400 *s*.

The ATRV-JR was equipped with a SICK LMS200 (for robot localization), an electronic nose and a ppbRAE 3000 PID. The electronic nose comprises four commercially available *Taguchi-type* (*TGS*) MOX gas sensors enclosed in an acrylic box and connected to the PID's suction pump through a plastic tube. The electronic nose is horizontally mounted behind the front bumper at a height of 0.1 *m*. The sensing configurations for both robotic platforms are listed in Table 1.

### 5.1. Sensor Selection

Sensor selection aims to find a subset *d̂* of the *D* available sensors in the array, that provides the best classification performance. Sensor selection is computationally expensive since, in order to find *d̂* by, e.g., K-fold cross validation (CV), it would be required to train and test $\sum_{d^{\prime }=1}^{D}K\times \left(\begin{array}{l} D\\ d^{\prime } \end{array}\right)$ classifiers. A less demanding alternative is to measure the class separability of the training data. Muezzinoglu and co-authors proposed to use the Mahalabonis distance (MD) as a separability metric to select an optimal heater temperature in a gas discrimination problem [41] with MOX sensors. The MD is proportional to the distance between-class centers and inversely proportional to the individual covariances. For normally distributed data, MD is the optimal estimator of the class overlap.

The MD is a valid separability index as long as the distributions under comparison have the same dimensionality. In the case of sensor selection, the subsets *d*′ can have different number of elements and thus, MD cannot be used straightforwardly. The solution we use instead is to compute the MD over the first components of the of the Principal Components Analysis projection [38] of *d*′. The number of principal components used in the computation of MD can be set according to the percentage of the captured variance. In this work, the MD is computed over the first three principal components, which capture at least 90% of the percent variance, and the optimal *d̂* is determined as follows:

(8) $$\hat{d}=\underset{d^{\prime }\subset D}{\arg\max}\sqrt{{\left(\mu_{1}^{\left(d^{\prime }\right)}-\mu_{2}^{\left(d^{\prime }\right)}\right)}^{T}S_{12}^{\left(d^{\prime }\right)}(\mu_{1}^{\left(d^{\prime }\right)}-\mu_{2}^{\left(d^{\prime }\right)})},$$

where $\mu_{1}^{\left(d^{\prime }\right)}$ and $\mu_{2}^{\left(d^{\prime }\right)}$ are the class centers and $S_{12}^{\left(d^{\prime }\right)}$ is the average of the covariances of each class (*i.e.*, pooled covariance matrix).

### 5.2. Parameter Selection

As shown in Equation (3a), the correction factors ξ for the target analytes have to be included in the computation of the gas distribution maps. According to the PID manufacturer (http://www.raesystems.com/customer-care/resource-center/tech-notes), the correction factors for ethanol, propanol and acetone are 9.6, 5.5 and 0.9 respectively.

In addition, there are two functional parameters that have to be optimized, namely the number of Gaussians γ in the classification algorithm and the bandwidth σ of the MC Kernel DM+V algorithm.

For the classification stage, an optimal *γ̂* is selected from a search space γ = [γ_1_, …,γ*_m_*] using K fold CV and likelihood maximization as shown in the following equations:

(9a) $$L(\gamma_{j}|\text{r})=\sum_{i=1}^{n}\;[l_{1}\log\left(p(l_{1}|\gamma_{j},\text{r})\right)+l_{2}\log\left(p(l_{2}|\gamma_{j},\text{r})\right)]$$

(9b) $$\hat{\gamma }=\underset{\gamma_{j}\in \gamma }{\arg\max}L(\gamma_{j}|\text{r})$$

where *l*_1_ and *l*_2_ are binary ground truth labels and and *p*(*l*_1_|γ*_i_*, **r**) and *p*(*l*_2_|γ*_i_*, **r**) are the estimated class posteriors. The ground truth labels are available, since parameter selection for the classifier is performed with data collected in the single source experiments listed in Table 1.

Similarly, for GDM, the optimal value of the parameter σ̂ is selected from a search space **σ** = [σ_1_,…,σ*_j_*]. In single compound GDM, σ̂ is learned by evaluating the capability of the model to predict unseen concentration measurements [17]. However, when multiple chemical compounds are present in the environment, as in the dual source experiments carried out in this work, this approach is not feasible due to the lack of ground truth with respect to the concentration values for each class. In order to collect a ground truth dataset, it would be required to have perfectly selective sensors to measure the gas concentration levels for each of the target compounds. This is hardly feasible in practice.

Instead we propose to learn the mapping parameters by evaluating the capability of the classification maps to predict the class posteriors of unseen measurements using the Total Variation Distance (TVD) [42] as a metric. The TVD quantifies the distance between two probability distributions, namely the class posteriors predicted by the classifier and the class predictions from the classification maps *γ_σ_j__*,*l*, computed with a kernel bandwidth σ*_j_*. The selection of TVD as a metric instead of, e.g., the more commonly used Kullback-Leibler (KL) divergence [38] is due to the fact that when the posteriors *p*(*l*|**r**) are close or equal to zero, the KL divergence is undetermined and thus assumed to be equal to 0. This means that a considerable amount of measurement points would have to be ignored. Using the TVD, the optimal σ̂ is selected as follows:

(10a) $$\text{TVD}(\sigma_{j})=\sum_{l=1}^{L}\sum_{i=1}^{n}\left|\lambda_{\sigma_{j},l}({\text{x}}_{i})-p(l|{\text{r}}_{i})\right|$$

(10b) $$\hat{\sigma }=\underset{\sigma_{j}\in \sigma }{\arg\min}\text{TVD}(\sigma_{j})$$

## 6. Results

The data collected in the single source experiments were used to optimize the parameters since for each experiment, the identity of the gas is known and therefore, ground truth is available to compute the classifier's performance. The trials in the robot arena and in the outdoor courtyard were carried out with different sensor sets and with different combinations of target substances (see Table 1). Therefore, the optimization process for the classifier was run separately for each scenario. The optimal number of Gaussians *γ̂* and the optimal sensor combination *d̂* were obtained using five fold CV with a search space ***γ*** = [2,3,…, 12]. For the robot arena, the classification success rate was 0.98 ± 0.07%, given by *d̂* = [*E2V* − *5135*, *E2V* − *2710*, *E2V* − *5521*] and *γ̂* = 5. In the outdoor courtyard, the success rate was 0.96 ± 0.01%, with *d̂* = [*TGS* − *2620*, *TGS* − *2602*, *TGS* − *2600*] and *γ̂* = 12.

**Figure 6.**
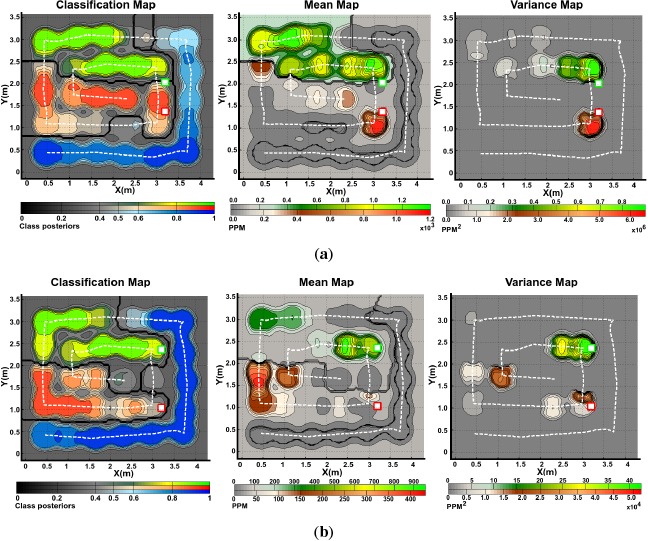
Generated gas distribution models of two experiments in the robot arena with Ethanol (green) and propanol (Red) gas sources separated by different distances **(a)** 0.5 *m*, **(b)** 1.5 *m*. The blue shades in the classification maps denote the likelihood of finding clean air at a given position in the explored area. The dashed lines denote the robot's path and the actual source locations are indicated by squared markers.

**Figure 7.**
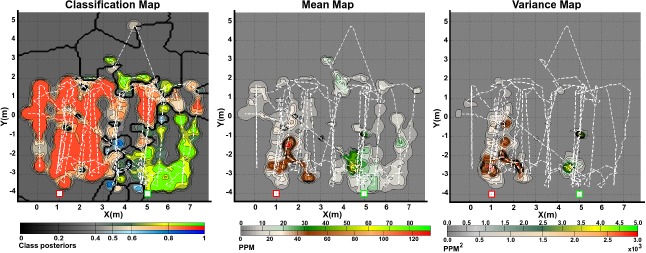
Generated gas distribution models in the outdoor experiment. In all the maps, green shades corresponds to ethanol and red shades correspond to acetone. The blue shades in the classification maps denote the likelihood of finding clean air at a given position in the explored area. The dashed lines denote the robot's path and the actual source locations are indicated by squared markers.

The obtained gas distribution models can be seen in Figures 6 and 7. Only two trials with different spacing between sources, for the robot arena, are shown in the figures. The kernel bandwidth σ for each experiment was learned using the proposed TVD approach. For all experiments, the bandwidth search space was set to **σ** = [0.05, 0.1,0.15,…, 2.00] and, for the experiments presented in Figures 6 and 7, the optimal kernel bandwidths σ̂ were 0.15, 0.15 and 0.20 respectively.

The classification map is presented in the form of a maximum a posteriori plot. The maps show higher probabilities of detecting a given analyte at locations where neighboring data samples were consistently classified as *l* with high confidence. In the case of Figure 6b, it can be noticed in the classification map that the Propanol likelihood drops close to 50% in the neighbouring locations around the gas source, while for locations away from it, the likelihood drastically rises up to 100%. Intuitively, high posteriors would be expected close to the actual gas source. However, due to the low concentration levels recorded by the robot nearby the source, the confidence in the predictions is low.

The predictive mean concentration maps were generated by combining the individual mean concentration maps for each substance masked with the classification maps. While we do not have ground truth to evaluate the accuracy of the distribution maps, it can be noticed that the computed distribution maps predict well defined plumes at the neighboring locations of the actual source locations. In a similar way as with the mean distribution maps, combined variance maps were generated as shown in the figures. We can intuitively evaluate the produced models by considering the implicit information they contain. It can be noticed that areas with high variance are located in the vicinity of the actual gas source locations, which is an expected result since, according to [22], concentration fluctuations are expected to peak significantly near an emitting gas source.

## 7. Conclusions

One of the most restrictive assumptions made by state of the art GDM algorithms is that only one chemical compound is present. This assumption rarely holds for real world scenarios, where tasks such as gas monitoring have to be carried out. In this paper, we address GDM of multiple compounds using the MC Kernel DM+V, which is a *model free* algorithm that removes the assumption of a single chemical compound. MC Kernel DM+V considers class posteriors from an external classifier and concentration measurements from a non specific sensor, such as a PID. In this way, MC Kernel DM+V produces independent true gas distribution models, one for each analyte and a set of classification maps, that predict the likelihood of detecting a given compound at a query location in the explored area. MC Kernel DM+V does not make any restriction regarding the technology or measuring principles of the sensors used, as long as point concentration measurements and estimations of the class posteriors are provided to the algorithm.

This paper presents several improvements on the original MC Kernel DM+V algorithm [7]. Rather than using the class posteriors in the subsequent computation of the mean and variance maps, in this work, we use the predictions of the classification maps. In this way, the spatial distribution of the predicted class posteriors is incorporated and erroneous predictions of the gas discrimination algorithm tend to be filtered out and they do not contribute to further computations. Second, we explicitly incorporated the correction factors of the PID sensor in the algorithm. The correction factors are provided by the sensor manufacturer for the quantification of different chemical compounds. By incorporating the correction factors, the predictions made by the maps can be considered as calibrated concentration values. Third, a novel algorithm to learn the kernel bandwidth for multi class gas distribution maps was presented. This algorithm aims to minimize the total variation distance between the predictions made by the classification map and the class posteriors drawn by the gas identification algorithm. In addition, the proposed algorithms were evaluated in a more extensive way. Apart from experiments in the robot arena, further trials were conducted in an outdoor location where an alternative sensor configuration was used to sense two different chemical compounds.

One of the major challenges when designing algorithms for gas sensitive mobile robots is the lack of a significant number of experimental trials. Even when dedicated experimental locations can be built (as the robot arena used in this work), there are several aspects that complicate the data collection process. First, gas dispersion is a complex phenomenon that depends on different environmental and topological conditions. Slight changes in e.g., temperature or wind flow conditions can substantially affect the outcome of the experiments and thus, repeatability between experiments is hard to achieve. Second, there are several practical aspects to consider when performing experiments, for example, the limited battery life of the robotic platforms, the drift of the gas sensors caused by aging/changing environmental conditions and the fact that, once a trial has been completed, the preparations to conduct a new experiment are time consuming. For example, indoor locations have to be ventilated in order to remove gas concentrations accumulated from the previous experiments, the gaseous sources have to be prepared and the robot's batteries have to recharged. In addition, when performing experiments with multiple chemical compounds present at the same time, it is hard to collect ground truth to perform quantitative evaluations w.r.t. the concentration level and the identity of the acquired measurements.

However, the experiments conducted in this work allowed to evaluate the proposed MC Kernel DM+V under environmental conditions that could be expected in practical applications. While the predictive capabilities of the gas distribution maps were not quantitatively evaluated, the obtained results show consistent distribution maps (in indoor and outdoor scenarios) where plume shaped structures predict high concentration areas near the measurement points where a high average concentration was sensed. In addition, it was observed that the variance maps can provide useful information by highlighting areas near the gas source locations.

In addition, it was observed that the selection of the gas discrimination algorithm, for the particular application of multi compound GDM, plays a crucial role. The gas discrimination algorithm used in this work (originally presented in [6]) allows for learning of a rejection class that denotes highly diluted measurements. By providing a class posterior for the rejection class, it is then possible to model areas in the exploration area where it can be assumed that no chemicals are present and thus, clean air can be assumed.

There are several directions that can be explored as future work. First, the algorithm can be extended to allow for multiple kernel bandwidths, one for each identified compound. The challenge here would be that the computational complexity of the parameter selection process will increase.

Second, gas mixtures can be considered in the computation of the distribution maps. For the specific case of a set-up where concentration measurements are acquired with a PID, the introduction of gas mixtures would not require further changes in the algorithm. This is due to the fact that the correction factors for gas mixtures can be computed as a linear combination of the individual correction factors for each identified compound. In this context, the key challenge would be to train a regression algorithm that computes the gas mixture percentage with the corresponding uncertainty estimation.

Third, a numerical evaluation of the effects caused by the sensors' time constants in multi compound GDM is needed. For single compound GDM, it has been reported that the sensors' time constant introduces a *memory effect* that produces shifting and blurring effects when computing gas distribution maps [20]. Due to the fast response times of the PID used in this work (≤ 3 *s*, according to the manufacturer) and the achieved average robot speeds in both scenarios ( 0.04 *m*/*s* indoors, 0.12 *m*/*s* outdoors), artifacts introduced by the PID can be disregarded. However, the time constants of the MOX sensors used in the gas discrimination stage are considerably longer than those of the PID sensor. In the case of the gas discrimination algorithm used in this work, it can be expected that the slow decay time of the MOX sensors could translate into a correspondingly decreasing class posterior. This aspect needs to be further investigated.

Fourth, the gas distribution maps are given as time invariant structures. This means that in the computation of the maps, the time stamps of the acquired measurements are not considered and therefore, the mean and variance concentration predictions remain constant over time. Time dependent/multi compound gas distribution maps could be generated for example, by extending the Time Dependent (TD) Kernel DM+V, proposed in [43], to consider the presence of multiple chemical compounds.

## Figures and Tables

**Table 1.** Summary of the experimental configurations during the validation trials.

**Location**	**Robot**	**PID**	**OSS Sensors**	**Trials**
**Robot arena** (5 *m* × 5 *m* × 2 *m*)	P3-DX	MiniRAE lite	(1) E2V-2610 (1) E2V-2710 (2) E2V-5521 (1) E2V-5121 (1) E2V-5135	3 trials, single ethanol source 3 trials, single propanol source 3 trials, ethanol and propanol sources separated by 0.5 *m* 3 trials, ethanol and propanol sources separated by 1.5 *m*

**Outdoor courtyard** (9 *m* × 5 *m*)	ATRV-JR	MiniRAE lite	(1) TGS-2600 (1) TGS-2602 (1) TGS-2611 (1) TGS-2620	1 trial, single ethanol source 1 trial, single acetone source 1 trial, ethanol and acetone sources separated by 2.0 *m*
